# Cross-Tailoring Integrative Alcohol and Risky Sexual Behavior Feedback for College Students: Protocol for a Hybrid Type 1 Effectiveness-Implementation Trial

**DOI:** 10.2196/43986

**Published:** 2023-03-20

**Authors:** Anne E Ray, Eun-Young Mun, Melissa A Lewis, Dana M Litt, Jerod L Stapleton, Lin Tan, David B Buller, Zhengyang Zhou, Heather M Bush, Seth Himelhoch

**Affiliations:** 1 Department of Health, Behavior & Society College of Public Health University of Kentucky Lexington, KY United States; 2 Department of Health Behavior and Health Systems School of Public Health The University of North Texas Health Science Center at Fort Worth Fort Worth, TX United States; 3 Klein Buendel, Inc Golden, CO United States; 4 Department of Biostatistics and Epidemiology School of Public Health The University of North Texas Health Science Center at Fort Worth Fort Worth, TX United States; 5 Department of Biostatistics College of Public Health University of Kentucky Lexington, KY United States; 6 Department of Psychiatry College of Medicine University of Kentucky Lexington, KY United States

**Keywords:** alcohol-related risky sexual behavior, college students, cross-tailored dynamic feedback, effectiveness-implementation hybrid designs, personalized feedback intervention, underage drinking

## Abstract

**Background:**

Underage drinking and related risky sexual behavior (RSB) are major public health concerns on United States college campuses. Although technology-delivered personalized feedback interventions (PFIs) are considered a best practice for individual-level campus alcohol prevention, there is room for improving the effectiveness of this approach with regard to alcohol-related RSB.

**Objective:**

The aims of this study are to (1) evaluate the impact of a brief PFI that integrates content on alcohol use and RSB and is adapted to include a novel cross-tailored dynamic feedback (CDF) component for at-risk first-year college students and (2) identify implementation factors critical to the CDF’s success to facilitate future scale-up in campus settings.

**Methods:**

This study uses a hybrid type 1 effectiveness-implementation design and will be conducted in 3 phases. Phase 1 is a stakeholder-engaged PFI+CDF adaptation guided by focus groups and usability testing. In phase 2, 600 first-year college students who drink and are sexually active will be recruited from 2 sites (n=300 per site) to participate in a 4-group randomized controlled trial to examine the effectiveness of PFI+CDF in reducing alcohol-related RSB. Eligible participants will complete a baseline survey during the first week of the semester and follow-up surveys at 1, 2, 3, 6, and 13 months post baseline. Phase 3 is a qualitative evaluation with stakeholders to better understand relevant implementation factors.

**Results:**

Recruitment and enrollment for phase 1 began in January 2022. Recruitment for phases 2 and 3 is planned for the summer of 2023 and 2024, respectively. Upon collection of data, the effectiveness of PFI+CDF will be examined, and factors critical to implementation will be evaluated.

**Conclusions:**

This hybrid type 1 trial is designed to impact the field by testing an innovative adaptation that extends evidence-based alcohol programs to reduce alcohol-related RSB and provides insights related to implementation to bridge the gap between research and practice at the university level.

**Trial Registration:**

ClinicalTrials.gov NCT05011903; https://clinicaltrials.gov/ct2/show/NCT05011903

**International Registered Report Identifier (IRRID):**

DERR1-10.2196/43986

## Introduction

### Overview

Despite declines over the past several decades, underage drinking and related harm continue to be major public health concerns on United States college campuses [1-3]. National data show two-thirds of college students are current drinkers, 1 in 3 reports past-month heavy-episodic drinking (more than 5 drinks in a row), and 1 in 10 reports high-intensity drinking (more than 10 drinks in a row) [1]. Greater college student alcohol consumption and heavy drinking on a given day are linked to increased sexual activity and risky sexual behavior (RSB; eg, unprotected sex and unplanned hookups) [4-9]. Alcohol-related RSB puts college students at risk for negative health outcomes (eg, sexually transmitted infections and unplanned pregnancies) and is a pathway to sexual victimization and escalated drinking [10-14]. Notably, alcohol use is implicated in approximately half of college student sexual assaults [14,15]. Given the high rates of alcohol misuse in college and the association between alcohol use and RSB, prevention efforts that reduce alcohol-related RSB have been identified as a priority area in the National Institute on Alcohol Abuse and Alcoholism (NIAAA) Strategic Plan 2017-2021 [3].

The first weeks of college are referred to as the red zone with escalations in heavy drinking [16-18] and sexual assault [19-23]. Changes in the salience of social and achievement goals and stressors during the transition to college impact alcohol use during this period [24]. Students who drink heavily prior to college are likely to escalate drinking [25], and nondrinkers or light drinkers may consume heavy amounts on occasion, increasing their risk of experiencing serious consequences [26,27]. This coincides with changes in students’ sex-related cognitions and behaviors [8,28]. Thus, effective interventions for escalated alcohol use and RSB during this critical period of risk can result in high public health and clinical impacts [29].

### Technology-Delivered Personalized Feedback Interventions

NIAAA’s College Alcohol Intervention Matrix identifies technology-delivered personalized feedback interventions (PFIs) as a best practice for individual-level campus alcohol prevention [30]. PFIs typically involve a one-time, web-based assessment of an individual’s alcohol use behaviors, followed by the provision of a tailored profile based on the assessment of the individual’s drinking patterns and strategies to reduce harm [31-33]. The utilization of technology for assessment and feedback allows PFIs to retain key features of efficacious in-person brief motivational interventions without the high costs and need for trained personnel [34]. Many universities now opt for commercially developed PFIs for first-year students (eg, AlcoholEdu for College [35] and eCHECKUPTOGO [36]) [37-42].

Past work from the investigative team [32,43,44] highlights 2 important concerns with PFIs as widely implemented. First, a rather exclusive focus on the assessment and feedback of alcohol use means other health behaviors impacted by alcohol use, such as RSB, are rarely addressed [32]. To address this gap, a PFI with integrated alcohol and RSB content for sexually active, college-aged young adults who regularly drink was developed. The integrated PFI showed promise in reducing alcohol-related sexual outcomes [43]. Second, there is considerable heterogeneity in PFI effects across participants [45] and studies [44,46]. Further, PFIs tend to provide short-term intervention benefits but offer diminished protection to college students beyond 6 months [46,47]. The current intervention is designed to increase and extend the effects of the integrated alcohol and RSB PFI by incorporating repeated and dynamic assessment and feedback.

### Extending PFIs With Cross-Tailored Dynamic Feedback

The prevailing approach to technology-delivered PFIs is to provide a single, point-in-time snapshot of one’s behaviors and beliefs relative to others, analogous to a “between-person” comparison, which can create initial discrepancy and enhance motivation to change [48]. However, this feedback is limited to a one-time snapshot of individual differences in risk, does not capitalize on the beneficial effects of continued monitoring, and cannot capture dynamic changes in an individual’s beliefs or behavior in response to feedback. The proposed PFI and cross-tailored dynamic feedback (CDF) adaptation (PFI+CDF) leverages semester-long diary assessments (once a day for 4 days a week over 12 weeks) to iteratively refine (ie, optimize) initial feedback. The addition of CDF has the potential to amplify the effects of PFIs through the provision of feedback linked to a user’s personal, day-to-day trajectory of a target behavior. The integration of adaptive (“just-in-time”) and dynamic feedback is designed to bolster and sustain the initial impact of the PFI by creating a unique form of self-monitoring of “within-person” trajectories. In this way, positive behavior change can be acknowledged, and the participant can be redirected when less desirable behaviors are reported. The CDF adaptation of a PFI is intended to develop discrepancy regarding alcohol-related RSB, alcohol use, and other RSB; enhance motivation to change; and boost self-efficacy in one’s ability to change. To our knowledge, dynamic optimization of intervention content with rapid deployment and evaluation has never been attempted with alcohol-related RSB interventions.

### Designing for Dissemination With an Effectiveness-Implementation Hybrid Design

Over the past 3 decades, researchers have made strides to enhance brief alcohol intervention implementation through a number of key adaptations (eg, extending to different subpopulations [44], altering delivery modality [49], adding boosters [50-52], giving students a choice for intervention assignment [53], adding video content [54], and adding substance-free activities [55]). Despite these efforts, a significant proportion of college campuses do not implement evidence-based strategies [56-58]. Although PFIs are a popular choice among campuses that do implement such strategies, little is known regarding strategies they use to encourage stakeholder groups’ participation or how PFIs complement other student health efforts.

Accordingly, the current intervention study is guided by a hybrid type 1 effectiveness-implementation design to examine PFI+CDF effectiveness and related implementation factors to improve and accelerate translation. Effectiveness-implementation hybrid designs offer a dual focus on both outcomes and process, which can serve to speed up the translation of an intervention if it is effective [59,60]. Process-oriented formative evaluations will be used with stakeholders at multiple levels prior to, during, and after program implementation to better understand implementation context, and barriers and facilitators to implementation [61-64]. It is critical to identify college-specific PFI implementation gaps at both the student level and systems level for the purpose of improving PFI+CDF’s acceptance by students and achieving greater adoption on campuses. By engaging multilevel stakeholders at the outset, a “designing for dissemination” approach is followed, using feedback from members of the population of users and organizational adopters [65-68]. In this paper, we provide a description of the proposed hybrid type 1 effectiveness-implementation study protocol.

## Methods

### Study Design

**Figure 1:**
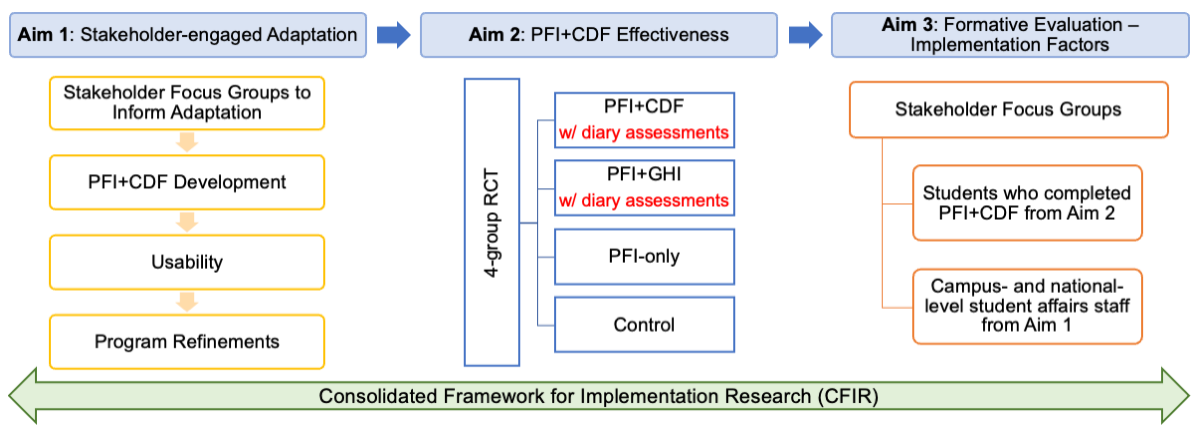
Overview of study design. CDF: cross-tailored dynamic feedback; GHI: generic health information; PFI: personalized feedback intervention; RCT: randomized controlled trial.

Figure 1 provides an overview of the 3-phase, multisite hybrid type 1 effectiveness-implementation design to evaluate the effectiveness and understand the implementation of a novel PFI+CDF to reduce first-year college students’ alcohol-related RSB. Phase 1 is a focus group-guided PFI+CDF adaptation and usability test. In phase 2, first-year college students who report past-month heavy-episodic drinking (more than 4 or 5 drinks for females or males within 2 hours) or drinking at least 3 times in the past month and who are sexually active without being in a monogamous relationship will be recruited from the University of Kentucky (UK) and the University of North Texas (UNT) to participate in a 4-group randomized controlled trial (RCT) to examine the effectiveness of PFI+CDF in reducing alcohol-related RSB. PFI+CDF will have 3 other comparison conditions: a PFI followed by the 12-week dynamic feedback on general health information (PFI+GHI), a PFI without any additional exposure to intervention (PFI), and assessment-only control (AOC). In phase 3, a qualitative evaluation will be conducted with multilevel stakeholders to better understand relevant implementation factors.

Phases 1 and 3 are guided by the Consolidated Framework for Implementation Research (CFIR), a comprehensive set of constructs describing factors influencing implementation across all stages of the implementation process [69,70]. CFIR is commonly used to guide formative investigations within the context of hybrid type 1 designs [61,64]. Constructs are grouped into 5 domains in the current intervention study: intervention characteristics (ie, characteristics of PFI+CDF), outer setting (ie, context surrounding student affairs), inner setting (ie, context within student affairs), individual characteristics (ie, students and student affairs staff), and process (ie, process for implementing PFI+CDF) [69].

### Adaptation (Phase 1)

#### Overview

Phase 1 will focus on adapting the existing integrated PFI to the enhanced PFI+CDF with the following steps: (1) focus groups with multilevel stakeholders, including students and student affairs staff; (2) building the data capture and content platform; (3) usability testing; and (4) program refinements. These 4 key iterative steps of adaptation are crucial in the systematic modification of existing evidence-based interventions to improve their effects and potential for implementation [66]. Furthermore, they ensure that the adaptation of the integrated PFI to PFI+CDF is achieved with the principle of designing for dissemination in mind [65].

#### Focus Groups

We will conduct focus groups with stakeholders (ie, students and student affairs personnel) to solicit their perspectives on a prototype of the proposed PFI+CDF intervention. Focus group participants will be students and student affairs professionals (eg, individuals involved with implementation of prevention programming) at UK and UNT, as well as student affairs professionals at the national level, recruited at the annual meeting of NASPA-Student Affairs Administrators in Higher Education, the premier conference in this field [71]. Recruitment and facilitation of focus groups at NASPA will ensure a national perspective on the PFI+CDF intervention.

At UK and UNT, we anticipate a single focus group for staff and 3 focus groups for students, with 5-8 participants per group (32 participants per site, 64 participants total). At NASPA, we will aim for 4 focus groups with 5-8 participants per group, for a total of 32 participants. Development of the focus group interview guide will be informed by the CFIR interview guide tool [72] and will target constructs within the intervention characteristics domain of CFIR (eg, adaptability, complexity, design quality, and relative advantage), exploring barriers and facilitators to implementation at both the student level and systems level [69,73]. Groups will last 60-90 minutes and incentives will be US $50 and US $100 gift cards for students and student affairs staff, respectively.

We will conduct a systematic thematic analysis to identify relevant themes from the qualitative data collections [74]. Focus groups will be transcribed and analyzed using NVivo [75]. A designated subset of the research team will first work independently to identify recurrent themes and important statements specific to intervention content. They will then review the independent coding together, discuss the independent coding with the entire research team, and come to a consensus about the emerging themes. The themes will be compiled into summaries and circulated to the research team to guide discussion of intervention refinement.

#### Data Capture and Content Development

##### Overview

Findings from the multilevel stakeholder focus groups will inform the development of an updated version of an existing evidence-based integrated alcohol and RSB feedback profile (PFI) and the addition of CDF content, as well as an assessment and delivery platform necessary to populate and house the PFI and CDF content.

##### PFI and CDF Delivery Platform

The PFI and CDF content will be housed in a website application platform that offers flexibility in reach and accessibility but is optimized for viewing across device types (eg, computer, tablet, and phone). For example, links to PFI and CDF content can be sent to participants via multiple channels (eg, SMS text message and email) to access feedback. The assessment and content platform will be built as an integrated system, essential for scale-up for several reasons: (1) survey responses inform both the PFI and CDF content and are a necessary part of the system, (2) it allows for a built-in evaluation mechanism, and (3) it creates one overall system that is portable for hosting at other sites. The underlying programming will be conducted in HTML5 to ensure flexibility for future adaptations to other platforms. The web-based app will run on common web browsers on iOS and Android smartphones, tablets, and on iOS and Windows personal computers. A backend tracking program will record user progress.

##### Intervention Content: PFI

Consistent with prior work, PFI content will be generated from responses to a baseline assessment and offer participants integrated feedback on both alcohol use and RSB across the following content domains: a behavioral profile, normative comparisons, blood alcohol content, expectancies, perceived risk, likelihood of engaging in future risk behaviors, and protective behavioral strategies. In addition to integrated alcohol and RSB content, the PFI will include an option to view feedback on alcohol use behaviors only, as pilot data indicated separate alcohol-focused content was necessary to reduce alcohol use not specific to sexual behavior.

##### Intervention Content: CDF

CDF content will be generated from weekly diary-style surveys (Friday to Monday) in which students report their alcohol use and RSB, as well as their preferences when interacting with CDF content. Participants will receive dynamic feedback on their own behaviors for 12 weeks of the fall semester, allowing them to see their behaviors over time and receive feedback on their behavior changes (ie, “within-person” feedback). Although the overall system and specific design features will be created in consultation with students and technology developers, and subsequently refined following the usability testing, it is anticipated that the CDF content will include (1) a brief and updated behavior profile, (2) normative feedback on their current weekly drinking and related sexual behavior pattern, (3) positive reinforcement for improving behaviors within weekly and monthly time frames in figures, (4) brief updated intervention content topics (eg, individual-specific protective strategies that they can use in the following week) along with an option to see a full version, and (5) alerts for potential areas for improvement and referral information, if needed.

#### Usability Testing and Program Refinement

Usability testing will focus on (1) soliciting feedback on users’ experience with the enhanced PFI+CDF and (2) troubleshooting the CDF diary surveys and dynamic weekly feedback methodology. The usability test will be conducted with the methodology, assessments, and timing of the RCT procedures for the PFI+CDF group (see phase 2 description below), with the exception that the testing does not need to start at the beginning of the academic year or include follow-up surveys. Participants (n=100, 50 per site) will provide standard ratings of the acceptability [76] (eg, attractiveness, comprehension, relevance, and persuasive value) and usability [77] (eg, ease and efficiency of use) of the PFI+CDF intervention and respond to open-ended questions regarding most and least liked features. We will create a summary of participants’ impressions, commonly referenced problems, and acceptability and usability ratings, and use this information for one last round of program refinement, as recommended in systematic adaptation approaches.

### Effectiveness Trial (Phase 2)

#### Overview of RCT Design

**Figure 2:**
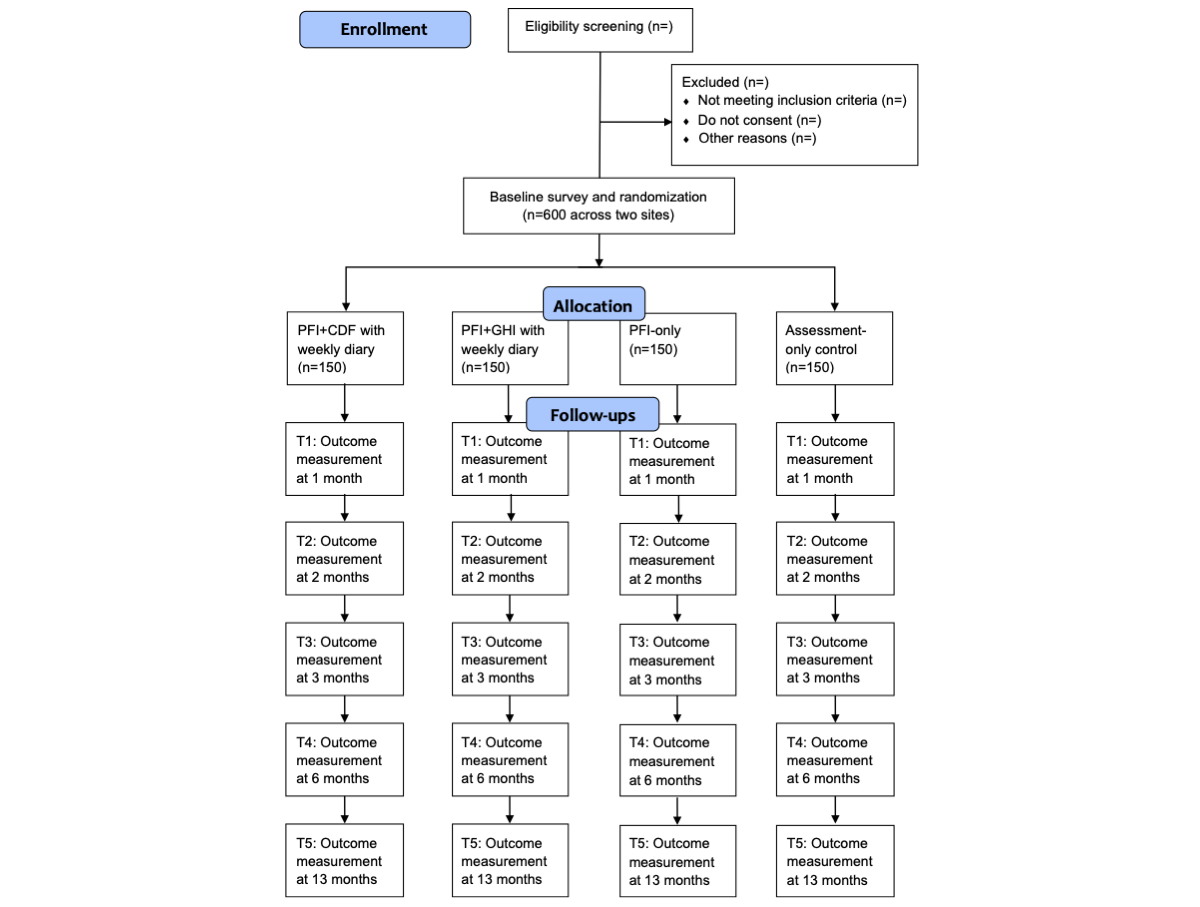
Randomized controlled trial workflow diagram. CDF: cross-tailored dynamic feedback; GHI: generic health information; PFI: personalized feedback intervention.

We will conduct a multisite, 4-group RCT to evaluate the intervention (Figure 2). Participants (N=600 total, 300 per site) will be randomized to 1 of 4 groups: (1) PFI+CDF with weekly diary surveys; (2) PFI+GHI (generic health information) with weekly diary surveys; (3) PFI-only, no weekly diary surveys; and (4) AOC, no weekly diary surveys. All participants will complete a baseline survey during the first week of the semester, be randomly assigned to 1 of the 4 groups, and complete follow-up surveys at 1, 2, 3, 6, and 13 months post baseline. The staggered design allows for comparison of the enhanced PFI+CDF relative to the PFI+GHI, which may be consistent with a “treatment-as-usual” comparison group (eg, of the universities that have adopted an evidence-guided alcohol intervention program for their campus, many currently deliver commercialized alcohol-focused PFIs to incoming first-year students) [35,36]. Providing weekly GHI in the comparison group allows for an equal number of “exposures” between PFI+CDF and PFI+GHI, analogous to an attention control group, offering a clearer understanding of the overall impact of the PFI+CDF adaptation. The inclusion of 2 PFI conditions, 1 with weekly diary assessments and 1 without, allows us to control potential assessment reactivity that might result from the diary-style assessment approach. This design is intended to allow a separation of the “true” intervention effect of the CDF above and beyond the effect of assessment reactivity. The PFI-only versus AOC group comparison will provide a test of the integrated PFI that has been adapted based on stakeholder feedback.

#### Recruitment

Contact information for a random sample of first-year college students between the ages of 18-20 years will be drawn from the registrar’s office at each study site. Students will be sent a prenotification letter on the university letterhead describing the study and inviting their participation. The letter will inform them to check their email on the study start date for more details and be followed by an email that reiterates study details, invites their participation, and includes a web link and personal identification number to access a web-based consent form and screening survey. Other recruitment methods will supplement this recruitment from the registrar’s office, including direct emails to interested students, advertisements in newspapers, on websites and social media, and postcards and fliers distributed at various on-campus locations. Eligibility criteria are shown in Textbox 1. Eligible students will be required to give informed consent before being directed to the baseline survey. Those who decline to consent and those who are not eligible will be directed out of the survey and removed from the participant list. Students will receive email reminders over the course of the following week. We will oversample underrepresented groups (n=300, 50% non-White and n=300, 50% White students) and maintain an equal ratio of men and women at each site.

**Eligibility criteria for the randomized controlled trial.**

**Eligibility criteria**

- First-year student
- 18-20 years of age
- Past 30-day heavy-episodic drinking (more than 4 or 5 drinks for females or males within 2 hours) or 3 or more drinking occasions within the past month
- Sexually active (oral, anal, or vaginal sex within past 12 months)
- Not in a monogamous relationship (single, not dating, or dating, not serious) or in a monogamous relationship (dating only 1 person) for less than 3 months or in a monogamous relationship but open to cheating
- Not pregnant or planning to become pregnant

#### Procedures

Following the baseline survey, participants randomized to 1 of the 3 PFI conditions will immediately be linked to their PFI, which will also be sent via email and SMS text message. A 2-week window is allotted at the beginning of the semester for participants to complete their baseline assessment and, if applicable, view their PFI content. Following that period, participants in the PFI+CDF and PFI+GHI will be sent prompts via email and SMS text message to complete diary surveys on Friday through Monday of each week. On Tuesdays, participants in the PFI+CDF group will receive a link to access their CDF corresponding to that week’s behavior, via text and email, and those in the PFI+GHI group will receive a link to access health topics relevant to college students guided by the American College Health Association website (eg, nutrition, mono, exercise, and immunizations) [78]. These procedures will continue for a total of 12 weeks. All participants will be asked to complete web-based 1-, 2-, 3-, 6-, and 13-month follow-up assessments and receive up to US $100 (US $15 per survey, with an additional US $10 bonus for the 13-month follow-up). Participants in the diary conditions will receive an additional payment up to US $96 (US $2 per day×4 days×12 weeks=US $96).

#### Intervention Assessments and Other Measures

Table 1 shows a list of self-report measures, including primary and secondary outcomes, demographics, and covariates. Primary and secondary outcomes and content-specific measures will be assessed at baseline and in all follow-up surveys. Measures will be adapted for weekly diary assessments along with items related to daily drinking context, consistent with our prior work. We anticipate baseline and follow-up surveys to be approximately 20 minutes in length, and daily surveys will be 5 minutes in length. Participants will be made aware of the importance of the data, the confidential nature of their responses, and we will follow procedures to secure their privacy.

**Table 1:** Self-report measures.

Construct		Description
**Primary outcomes**		
	Alcohol-related sexual behavior	Number of times that alcohol was consumed during or before sex, quantity of alcohol consumed before sex [79]
	Alcohol-related consequences—sexual	Sexual consequence scale [80]
**Secondary outcomes**		
	Alcohol use behavior	Daily drinking questionnaire [81], heavy-episodic drinking, and high-intensity drinking
	Alcohol-related consequences—nonsexual	Young adult alcohol consequences questionnaire [82]
	Sexual behavior	Frequency of sex (including oral, vaginal, or anal), number of casual sexual partners, and number of times that a condom was used during sex [79,83]
**Demographics and covariates**		
	Background demographics	Birth sex, gender identity, sexual orientation, race and ethnicity, height and weight (for blood alcohol content calculation)
	Alcohol use history	Lifetime alcohol use, age of drinking and intoxication onset, and family history
	Sex history	Age of first sex and history of sexual behavior

In addition to self-report measures, participant behavior analytics will be captured to help ensure intervention fidelity and be included as covariates in analyses. Analytics include (1) whether or not participants accessed their PFI, CDF, or GHI; (2) the number of times each PFI, CDF, or GHI was accessed; (3) time spent viewing each PFI, CDF, or GHI; and (4) time spent viewing each topic area within the PFI, CDF, or GHI. These data will be captured through tracking routines programmed on the website. We will examine the depth of college students’ engagement with intervention content to assess the effects of program exposure (ie, dose) based on the level of access to content (eg, ranging from students who looked at PFIs and all weekly CDF and GHI content vs those who engaged in some or none of the content) and time spent on various intervention components. These data will provide intervention acceptability metrics and allow us to control each participant’s exposure to the PFI, CDF, or GHI content in outcome analyses.

#### Sample Size Justification

Power analyses to detect intervention main effects were conducted using Optimal Design Plus Version 3.01 software and the “power determination approach.” Based on past research, we assumed a standardized effect size of 0.3 (a small to medium effect size) from the repeated measures RCT with 5 follow-ups over 13 months. To examine the effect of the PFI+CDF and PFI interventions, compared to the control, a minimum of 452 participants are required for a power of 0.8 at an alpha level of .05. To examine the effect of the PFI+CDF intervention (vs PFI+GHI or PFI-only intervention), at least 350 participants are required. Based on this power analysis, there is sufficient power (≥0.89) to detect a small to medium effect size for the integrated interventions (PFI+CDF, PFI+GHI, and PFI only) versus control and sufficient power to detect the small effect of the PFI+CDF intervention compared to the PFI+GHI or PFI-only intervention over time. Although the overall effect size was conservatively assumed to fall between small and medium in size, due to the fine-grained assessment approach within the time period where most changes are likely to occur, followed by a long-term 13-month follow-up assessment, we anticipate that the proposed study is sufficiently powered.

#### Statistical Analysis

For study phase 2, we will use a multilevel model for the main outcome analysis where the participant is defined at the highest level of the model (level 2), and observations (level 1) are nested within the participant. We will assume that the observation-level (level 1) residual error term and participant-level (level 2) intercept coefficients (ie, random effects) are each normally distributed with mean zero and a variance that is estimated from the data, while intercept and slope coefficients are multivariate normally distributed with mean zero for each, and a covariance matrix that is estimated from the data. Intervention groups will be tested as a fixed effect. Given that many outcomes of our interest are count outcomes with excessive zeros, we will utilize appropriate models to accommodate a zero-inflated outcome distribution with overdispersion [44,84]. The 2 sites can be entered as a random effect (level 3) or a fixed effect in a 2-level model. We will include an interaction term to test whether PFI+CDF is similarly effective for men and women. The intercept and growth slopes will be appropriately specified to maximize information.

We will use a parallel process growth model to test the codevelopment of correlated processes (eg, alcohol use and RSB) and a parallel process growth mediation model. This allows for the examination of 2 potentially dependent reduction trajectories, which are altered by intervention. We expect that those who reduce alcohol misuse more rapidly in response to intervention will also exhibit a trajectory in which one’s likelihood of engaging in RSB decreases, which can be tested by comparing a model where the 2 slope terms are correlated with a null model where its correlation is constrained to be zero. Intervention effects can be explicitly tested by adding intervention membership as an external covariate that changes these coupled processes. Intervention effect modifiers or moderators will be tested in a confirmatory (eg, sex) or exploratory investigations.

### Implementation (Phase 3)

A qualitative postintervention assessment of intervention implementation will be conducted with multilevel stakeholders, including students and both campus- and national-level student affairs staff. The goals are to better understand (1) participating students’ experiences with the intervention; (2) barriers and facilitators to implementation, guided by CFIR; (3) potential program sustainability; and (4) necessary modifications for other sites for scale-up.

Focus group participants will be a subset of students from the PFI+CDF arm of the RCT, as well as campus- and national-level stakeholders who also participated in preimplementation focus groups. Participants in the PFI+CDF condition will be asked if they are interested in participating in a follow-up study after the 13-month follow-up. We will randomly select up to 48 students who indicate interest in participating (n=24 per site) at varying levels of engagement with the intervention (as guided by program analytic measures) for a maximum of 3 focus groups per site (5-8 participants per group). Campus student affairs staff will be contacted via phone and email for staff focus groups (1 focus group per site for student affairs staff with 5-8 participants per group). At the national level, we will conduct 4 focus groups, with 5-8 participants per group, yielding a total of up to 32 participants. All focus groups will last 60-90 minutes with US $50 and US $100 gift card incentives for students and staff, respectively. The analytic plan for phase 3 qualitative data will mirror phase 1.

### Ethics Approval

The study underwent review and approval by the UK Institutional Review Board (IRB #62769), which serves as the single IRB of record for this study. All participants will sign an approved consent form in accordance with the ethical standards of Helsinki.

## Results

Recruitment and enrollment for phase 1 began in January 2022. The findings of phase 1 will inform the development and build of integrated PFI+CDF. It is anticipated that phase 2 will begin in August 2023, and phase 3 will begin in October 2024. Data will be shared in the NIAAA Data Archive twice a year (April 1 and October 1), starting from October 1, 2023. Findings will be published in peer-reviewed journals and presented at international, national, or regional professional meetings and conferences.

## Discussion

### Overview

This project aims to fill a gap highlighted in the NIAAA Strategic Plan 2017-2021 [3] by advancing an intervention that integrates alcohol misuse and RSB. It also directly addresses the 2 primary areas of foci underlying a recent NIAAA workshop to advance the science on evidence-based brief interventions for young adult populations: (1) optimizing intervention design and (2) implementation and scale-up [85]. To the best of our knowledge, the use of dynamic feedback based on individual behavior trajectories to optimize PFI content with rapid and sustained deliveries and ongoing evaluations has never been attempted with this population and area of study. Understanding the impact of a dual-risk behavior PFI that incorporates novel, cross-tailored between- and within-person feedback profiles can help inform best practices for the delivery of prevention interventions relevant to college students. If successful, our approach could influence how PFIs are developed and delivered in other populations and settings for maximum benefit. In addition, employment of a hybrid type 1 effectiveness-implementation design for this population, behavior, and campus setting is also the first of its kind and is expected to yield broadly informative outcomes through its rigorous randomized trial design testing intervention effectiveness and multilevel stakeholder engagement pre- and postimplementation.

### Limitations

There are several potential limitations that need to be acknowledged. First, the emphasis on first-year college students may be restrictive. We chose to focus on first-year students because they are at risk for alcohol use and RSB [16-26], but older students also experience alcohol-related harms. Many college campuses require students to complete a brief alcohol intervention at the start of college and often use existing commercialized PFIs, providing a built-in audience for the adapted PFI+CDF intervention [35,36]. We encourage future studies to explore PFI+CDF in other at-risk populations, such as older students and young adults not in college. Second, the PFI+CDF intervention approach is intensive and could lead to concerns about student engagement. However, our past work on integrated PFIs indicates that participants engaged with feedback content and viewed it multiple times [86]. We demonstrated that increased personalization leads to a stronger program effect, potentially mediated through increased attention to content [32]. By involving students in the design process in phase 1, we should enhance their interest [59,87,88]. Students also reported favorable reactions to the proposed study methods. Thus, we anticipate high levels of engagement, but will also examine program engagement in phase 2 analyses and phase 3 focus groups. Third, there are alternatives to a web-based app platform. Technology changes rapidly, with many other options for content delivery (eg, native mobile apps, smartwatches, social media). Our goal was to create a flexible system to deliver content in multiple ways (email and SMS text message), increasing the likelihood of engagement. Native mobile apps require more programming than web apps to operate across smartphone types and operating systems. Social media offers opportunities (eg, user-generated interaction and content) but also its own challenges (eg, spread of misinformation). Alternate delivery platforms will be explored during focus groups, and we anticipate the need for content to be portable to other platforms in future implementation.

### Conclusions

This innovative, multisite hybrid effectiveness-implementation study will (1) evaluate the impact of a PFI that integrates content on alcohol use and RSB and is adapted to include an innovative CDF component for at-risk first-year college students and (2) identify implementation factors critical to its success to facilitate future scale-up in campus settings. The findings of this study will improve existing evidence-based alcohol programs and bridge the gap between research and practice at the university level.

## Appendix

Multimedia Appendix 1 Peer-reviewer report from the Interventions to Prevent and Treat Addictions Study Section - Risk, Prevention and Health Behavior Integrated Review Group (National Institutes of Health, USA).

## Abbreviations

AOC: assessment-only control
CDF: cross-tailored dynamic feedback
CFIR: Consolidated Framework for Implementation Research
GHI: generic health information
IRB: Institutional Review Board
NIAAA: National Institute on Alcohol Abuse and Alcoholism
PFI: personalized feedback intervention
RCT: randomized controlled trial
RSB: risky sexual behavior
UK: University of Kentucky
UNT: University of North Texas

## Data Availability

Data sharing is not applicable to this paper as no data sets have been generated or analyzed. Once generated, this study will submit and share data with National Institute on Alcohol Abuse and Alcoholism (NIAAA) Data Archive, a data repository housed within the National Institute of Mental Health Data Archive (NDA).
