# Supplementary material for: Cross-Tailoring Integrative Alcohol and Risky Sexual Behavior Feedback for College Students: Protocol for a Hybrid Type 1 Effectiveness-Implementation Trial
Source: JMIR Res Protoc. 2023 Mar 20;12:e43986. doi: 10.2196/43986 (PMC10131715; doi:10.2196/43986)
Supplement: Multimedia Appendix 1 [file resprot_v12i1e43986_app1.pdf]

SUMMARY STATEMENT
(Privileged Communication)

PROGRAM CONTACT:
Dr. Robert Freeman
301443-8820
rfreeman@mail.nih.gov

Release Date: 06/15/2020
Revised Date:

Application Number: 1 R01 AA028246-01A1
Formerly: 1R01AA028246-01A1

Principal Investigator

RAY, ANNE ELIZABETH

Applicant Organization: UNIVERSITY OF KENTUCKY

Review Group: IPTA
Interventions to Prevent and Treat Addictions Study Section

Meeting Date: 06/04/2020
Council: OCT 2020
Requested Start: 09/01/2020
RFA/PA: PA18-390
PCC: AP F

Project Title: Cross-Tailoring Integrative Alcohol and Risky Sex Feedback for College Students: A Hybrid Type 1 Effectiveness-Implementation Trial
SRG Action: Impact Score:20 Percentile:3
Next Steps: Visit https://grants.nih.gov/grants/next_steps.htm
Human Subjects: 48-At time of award, restrictions will apply
Animal Subjects: 10-No live vertebrate animals involved for competing appl.
Gender: 1A-Both genders, scientifically acceptable
Minority: 1A-Minorities and non-minorities, scientifically acceptable
Age: 7A-Only Adults, scientifically acceptable

Project Year	Direct Costs Requested	Estimated Total Cost
1	408,673	629,268
2	498,996	768,345
3	497,202	765,583
4	495,597	763,112
5	449,476	692,095
TOTAL	2,349,944	3,618,403

ADMINISTRATIVE BUDGET NOTE: The budget shown is the requested budget and has not been adjusted to reflect any recommendations made by reviewers. If an award is planned, the costs will be calculated by Institute grants management staff based on the recommendations outlined below in the COMMITTEE BUDGET RECOMMENDATIONS section.
EARLY STAGE INVESTIGATOR
NEW INVESTIGATOR

1R01AA028246-01A1 Ray, Anne

EARLY STAGE INVESTIGATOR
NEW INVESTIGATOR
PROTECTION OF HUMAN SUBJECTS UNACCEPTABLE

RESUME AND SUMMARY OF DISCUSSION: This application proposes to develop and test a cross-tailored dynamic feedback intervention designed to enhance the effects of an integrated personalized feedback intervention for alcohol misuse and risky sexual behavior in college students. During the discussion, reviewers noted that the highly innovative use of a dynamic feedback system to address the significant problem of alcohol-linked sexual risk behavior has the potential to increase the short-term effects of personalized feedback interventions and advance intervention delivery in multiple fields. Significance is somewhat reduced, however, by the exclusion of naïve drinkers who escalate their drinking during the at-risk period. The highly responsive resubmission retains the rigorous multi-site, hybrid type 1 effectiveness-implementation design and methods, and has been improved by strengthening the rigor of the prior research in college students, better justifying the implementation aims, and including stakeholders other than students. The primary remaining concern with the approach is the lack of adequate information about participant flow to determine recruitment feasibility, particularly given the move to a new site. Overall, the panel concluded that the strengths of this innovative application outweigh the weaknesses, and the proposed project has the potential for high impact.

DESCRIPTION (provided by applicant): Alcohol misuse and related risky sexual behavior (RSB) are significant public health concerns among college students. Two-thirds of students are current drinkers, at least 1 in 3 report past month binge drinking (5+ drinks in a row), and 1 in 10 report high intensity drinking (10+ drinks in a row). Greater student alcohol consumption and heavy drinking on a given day is linked to increased sexual activity and RSB (e.g., unprotected sex, unplanned hook-ups). This puts students at risk for negative health outcomes (e.g., STIs) and is a pathway to sexual victimization and escalated drinking. The first few weeks of college, or the ‘red zone,’ present a critical window of opportunity to intervene for escalated alcohol use and associated risks, which can result in a potentially high public health and clinical impact. However, individual-level prevention strategies for college students tend to focus on students’ alcohol use patterns and consequences more broadly, with little to no integration of content on the relationship between alcohol use and RSB, an important gap in the literature and a priority area for NIAAA. Our team previously established the short-term efficacy of a personalized feedback intervention (PFI), a gold standard intervention approach, with integrated content on alcohol and RSB. We propose to extend our integrated PFI to include a cross-tailored dynamic feedback (CDF) component, which leverages technology to incorporate daily assessments of student behavior and provide users with dynamic weekly feedback over 12 weeks to amplify the effectiveness of the integrated PFI and to be easily implemented on college campuses. The project utilizes a multisite, hybrid type 1 effectiveness-implementation study design to (1) evaluate the impact of CDF for at-risk first-year college students and (2) identify implementation factors critical to its success to facilitate future scale-up in campus settings. The first aim is to conduct a multi-level stakeholder-engaged adaptation of the integrated alcohol and risky sex PFI through the development and inclusion of CDF. The second aim is to conduct a randomized controlled trial (RCT) of the enhanced intervention (PFI+CDF) in a sample of 600 first- year college students who report recent binge drinking and are sexually active. Our primary hypothesis is that participants who receive the PFI+CDF intervention will report less alcohol use, fewer risky sexual behaviors, and fewer consequences relative to those who receive a PFI supplemented with generic health information at follow- up (1, 2, 3, 6, and 13 months). Our third aim seeks to identify factors critical to PFI+CDF

implementation in campus settings through conducting focus groups with a subset of students from the RCT and with local and national systems-level stakeholders. The intervention has strong potential for widespread dissemination and targets a group at high risk for alcohol misuse and RSB.

PUBLIC HEALTH RELEVANCE: Alcohol misuse and related risky sexual behavior are significant public health concerns among college students. This project involves (1) adapting and evaluating a technology-delivered behavioral intervention that is designed to reduce alcohol misuse and related risky sexual behavior among first-year college students while also (2) examining factors important to intervention implementation in college settings. If effective, the intervention may be widely disseminated and has the potential to make a positive impact on college student health.

CRITIQUE 1

Significance: 1
Investigator(s): 2
Innovation: 2
Approach: 1
Environment: 2

Overall Impact: This is a R01 resubmission in response to FOA PA18-390. The application proposed to use a multisite, hybrid type 1 effectiveness-implementation study design to test an integrated (risky alcohol and risky sex) personalized feedback intervention with a cross-tailored dynamic feedback for risky college students at two sites. The first aim is to conduct focus groups with both students and student affairs stakeholders to adapt and refine the intervention. The second aim is a multi-site randomized controlled trial comparing four conditions that allow for the elucidation of self-monitoring and assessment effects. Outcomes include alcohol use, risky sexual behaviors, and fewer consequences at 1, 2, 3, 6, and 13-month follow-ups. The third aim involves focus groups with students as well as local and national student affairs stakeholders to examine implementation issues. This is a very well written application that is highly responsive to previous reviews in maintaining strengths and adapting. The potential public health impact of the project is high and the application provides a strong rationale for the current study based on a large body of rigorous research conducted by the team itself and the field in general. The investigative team is well selected to meet all of the aims of the grant and will provide extensive support to the early career PI. The approach to each aim is highly rigorous, with clear methodological and analytic plans.

1. Significance:

Strengths

- The application demonstrates the prevalence of unhealthy alcohol use among college students as well as the link between alcohol use and risky sexual behaviors, including event-level data.
- The application describes a body of rigorous research that supports the promise of BMIs, PFIs, integrated PFIs, and integrated PFIs that include dynamic feedback in college samples.
- The application describes the heterogeneity of PFI outcomes and provides a comprehensive review of others' and their own work that supports the promise of elements inherent in their intervention.
- The team developed and evaluated an integrated PFI with promising results.

- The application provides data supporting the acceptability of the intervention to college students based on focus groups and the feasibility of students accessing the feedback without incentives.
- It is compelling the application highlights how colleges face choices about adaptation of PFIs and we know little about how implementation decisions are made in this area.

Weaknesses

- None noted.

2. Investigator(s):

Strengths

- The PI has described her involvement in several clinical trials.
- The roles and responsibilities of Co-Is and consultants are clearly described
- Klein Buendel will oversee the development of the web-based program and has extensive track record in this department
- Faulty at the University of North Texas Health Science Center will assist in the development of the intervention and real time analysis of data
- There have been some shifts in Co-Is based on the PIs new appointment at UK, however these seem well justified.

Weaknesses

- The PI’s experience is limited to study management vs. leadership at the PI level.

3. Innovation:

Strengths

- To date, no trials have evaluated an integrated PFI with cross-tailored dynamic feedback.
- The use of a hybrid type 1 effectiveness-implementation study design is innovative.

Weaknesses

- None noted.

4. Approach:

Strengths

- The application provides a strong rationale for an HT1 design based on preliminary research supporting the promise of the intervention.
- Involvement of both students and other stakeholders such as campus administrators increases the relevance of Aims 1 and 3.
- It is compelling the application highlighted how colleges face choices about adaptation of PFIs and we know little about how implementation decisions are made in this area
- The application of the CFIR model to the focus groups in Aim 1 is clear.
- The approach is comprehensively described, rigorous, and appropriate to meet the aims of the grant.

- The analytic plan for each Aim is clear and rigorous.

Weaknesses

- None noted.

5. Environment:

Strengths

- The Department of Health, Behavior and Society within the University of Kentucky College of Public Health (CPH), University of North Texas Health Science Center (UNTHSC), and Klein Buendel (KB) have environments that are ideal to achieve the aims of the proposal.

Weaknesses

- None noted.

Study Timeline:

Strengths

- The timeline is detailed and appears feasible.

Weaknesses

- None noted.

Protections for Human Subjects:

Acceptable Risks and/or Adequate Protections
Data and Safety Monitoring Plan (Applicable for Clinical Trials Only):

Acceptable

Inclusion Plans:

- Sex/Gender: Distribution justified scientifically
- Race/Ethnicity: Distribution justified scientifically
- For NIH-Defined Phase III trials, Plans for valid design and analysis:
- Inclusion/Exclusion Based on Age: Distribution justified scientifically
- Adult participants 18-20 with gender and ethnicity/race characteristics representative of the campus populations. There is no justification for the exclusion of 17yo college students.

Vertebrate Animals:

Not Applicable (No Vertebrate Animals)

Biohazards:

Not Applicable (No Biohazards)

Resubmission:

- The application is highly responsive to prior review. The significance section has been updated to include data specific to college populations and event-level data and providing more rationale for implementation aims. Including stakeholders other than students in Aims 1 and 3 increases relevance.

Resource Sharing Plans:

Not Applicable (No Relevant Resources)

Budget and Period of Support:

Recommend as Requested

CRITIQUE 2

Significance: 3
Investigator(s): 1
Innovation: 1
Approach: 2
Environment: 1

Overall Impact: Alcohol use and risky sexual behavior are significant public health problems among college students that are both interrelated and associated with other negative health outcomes. The importance of effective and early intervention is thus paramount. The proposed work would adapt an integrated personalized feedback intervention (PFI) for alcohol use and risky sexual behavior to include a cross-tailored dynamic feedback (CDF) component, which is highly innovative and has strong potential to heighten impact via this precision medicine approach. There is strong evidence that PFI's reduce college student drinking, but effects are limited in duration and rarely target alcohol-related negative outcomes, such as risky sexual behavior (RSB). The proposed study would add to the scientific literature via support for the use of just-in-time, personalized approaches for the proposed outcomes as well as a broader range of alcohol-related risk behavior. The team is qualified to conduct the proposed work and the environment is strong. The rigor of the research design is strong and includes qualitative methods to inform the adaptation, dissemination, and implementation process. The overall significance of the project is somewhat dampened by the exclusion of naïve drinkers who escalate their drinking during the at-risk "red zone" period. Minor weakness to the approach are not score driving. In conclusion, the application's strengths significantly outweigh these weaknesses and result in very strong proposal with high potential impact.

1. Significance:

Strengths

- Personalized feedback interventions (PFIs) reduce college-student drinking, but the effects are limited in duration and rarely target alcohol-related negative outcomes, such as risky sexual behavior (RSB). The proposed integration of cross-tailored dynamic feedback to the research team's evidence-based, tech-delivered PFI for alcohol use and RSB has strong potential to address both gaps in the literature. [major strength]
- The proposed work is of key importance to the field in its innovative advancement of a personalized, dynamic approach to delivering intervention content. If successful, this work would

support the use of just-in-time, personalized approaches to reduce a broad range of alcohol-related risk behavior. [moderate strength]

- Delivering the intervention during the high-risk “red zone” period has high potential impact in the short-term but may also alter long-term trajectories of alcohol-related risk behavior. [minor strength]

Weaknesses

- The sample is limited to students who report at least one binge drinking episode in the 30 days prior to the first week of college. This may exclude a key subgroup of students who (as noted in the proposal) are at particular risk during the “red zone” period: those who are non- or light drinkers when they come to college (and have little experience judging their tolerance and/or navigating drinking situations) but then engage in occasional heavy or binge drinking during the red zone period. Thus, even if the aims are achieved, it is unclear that results would generalize to these students. [moderate weakness]
- The extent to which daily assessments are designed or able to capture temporal sequencing between alcohol use and RSB is unclear. Assessing this sequencing will provide data on alcohol use as an antecedent and consequence of RSB – which the proposed strategy does not seem to accomplish. It will also allow for tests of whether reduced alcohol use leads temporally to a lower likelihood of other primary outcomes, including outcomes not assessed with respect to alcohol use (e.g., sex without a condom). Such data seems important to work that presumes alcohol use is contributing to RSB. [minor weakness]

2. Investigator(s):

Strengths

- The PI has assembled a strong team that possesses all the expertise necessary to achieve the proposed aims. Numerous team members have a history of collaboration, which will be critical to the execution of a multi-site project with numerous moving parts. [major strength]
- The PI has a wealth of expertise for an early stage investigator that is relevant to the key aspects of the project (e.g., the study population, implementing and evaluating interventions at the university-level, adapting interventions for technology delivery) as well as expertise leading large research projects while at REAL Prevention, LLC. [major strength]

Weaknesses

- None noted.

3. Innovation:

Strengths

- The integration of personalized normative feedback with a cross-tailored dynamic feedback intervention represents an innovative shift in the field. [moderate strength]
- The use of machine learning technology to develop and deliver personalized feedback over a 3-month period is an innovative application in this field. This innovation allows for the novel augmentation of a widely used, but “static”, personalized normative feedback intervention with a precision medicine approach that heightens potential impact. [major strength]

Weaknesses

- None noted.

4. Approach:

Strengths

- The experimental design is well conceived and includes appropriate comparison groups as well as checks to account for intervention exposure and assessment reactivity. Overall, the study design will allow for a clearer understanding of the impact of the PFI+CDF adaptation. [major strength]
- Use of qualitative methods with stakeholders to inform adaptation, dissemination, and implementation process increases the potential impact. [moderate strength]
- Focus groups will include students and student affairs professionals, the latter of whom will reflect professionals at the RCT sites and at the national level. This will maximize the likelihood that the intervention can be adapted with broader dissemination as an end goal. [moderate strength]

Weaknesses

- Anticipated size of incoming freshman classes at each site are not mentioned. Thus, the likelihood of obtaining 300 eligible students from each site cannot be fully evaluated. Relatedly, there are not letters of support from each institution which support the research team's plan to obtain student contact information from the registrar's office. [minor weakness] This weakness is somewhat offset by Co-I Lewis and Bush's experience with this recruitment approach at their respective institutions (although Co-I Lewis only recently moved to UNT so it is unclear if the necessary relationships have been built).
- Sexual and gender minority students are at greater risk for heavy episodic drinking and related health consequences, including RSB, that is driven in part by sexual and gender minority stigma. While not a central focus of this proposal, not assessing sexual and gender minority identities prevents any consideration of how (1) the intervention may be experienced by these students (Aim 1, Aim 3) and (2) effective the intervention is for these students. [minor weakness]

5. Environment:

Strengths

- The research environments at the University of Kentucky, University of North Texas Health Science Center, and Klein Buendel all provide sufficient resources to support the proposed study procedures.

Weaknesses

- None noted.

Study Timeline:

Strengths

- Timeline is detailed and reasonable.

Weaknesses

- None noted.

Protections for Human Subjects:

Unacceptable Risks and/or Inadequate Protections

- All consent forms will include referral information to campus resources and national hotlines related to sexual violence victimization. For those who request referrals, the team will provide them. However, the daily monitoring methodology will result in the team knowing about most sexual victimization experiences within 24 hours of its occurrence, and the team has the capacity to screen for this on a daily basis (in the same manner that potentially lethal alcohol use is identified). However, no procedures are in place which address Title IX mandated reporting or follow-up contact with the participant regarding the victimization experience.

Data and Safety Monitoring Plan (Applicable for Clinical Trials Only):

Acceptable

- DSMP is appropriate including the use of a DSM board.

Inclusion Plans:

- Sex/Gender: Distribution justified scientifically
- Race/Ethnicity: Distribution justified scientifically
- For NIH-Defined Phase III trials, Plans for valid design and analysis: Scientifically acceptable
- Inclusion/Exclusion Based on Age: Distribution justified scientifically
- Distribution of sex/gender and race/ethnicity are expected to reflect the college population at the two universities. Children are excluded as the because it is unlikely that there is large number of children at these universities.

Vertebrate Animals:

Not Applicable (No Vertebrate Animals)

Biohazards:

Not Applicable (No Biohazards)

Resubmission:

- The application is highly responsive to prior review comments. The rigor of prior research has been strengthened in several areas, the justification for the implementation framework is strong, the PI's expertise and experience managing large, multisite NIH trials has been clarified, and relevant pilot data has been provided regarding the acceptability and feasibility of PFI+CDF.

Resource Sharing Plans:

Acceptable

Budget and Period of Support:

Recommend as Requested

CRITIQUE 3

Significance: 2
Investigator(s): 2
Innovation: 1
Approach: 4
Environment: 2

Overall Impact: The proposed R01 is a resubmission of a two-site effectiveness-implementation study evaluating the impact of cross-tailored dynamic feedback (CDF) for heavy-drinking first-year college students who are sexually active. It involves adapting an efficacious, integrated PFI for alcohol and sexual risk behavior based on focus groups with stakeholders; examining the effectiveness of the PFI+CDF among at-risk college students; and evaluating factors that influence implementation, again via focus groups. The potential impact of this research is high, and the use of dynamic feedback is innovative. The research team is somewhat large; however, each investigator is strong in their respective area. The research design is innovative for the alcohol and addiction literature and is expected to improve the scalability of the intervention. Investigators were responsive to previous research concerns. The primary remaining weaknesses of the proposal is inadequate description of participant flow to determine feasibility of recruitment.

1. Significance:

Strengths

- Greater alcohol consumption is linked to risky sexual behavior at the within-person level.
- Risky sexual behavior has been associated with STI, unplanned pregnancy, and sexual victimization.
- The first few weeks of college are associated with escalations in both drinking and sexual assault.
- Integrated alcohol and SRB interventions are needed because alcohol intoxication and related contextual factors play a central role in sexual decision-making.
- The hybrid effectiveness-implementation design will speed translation of scientific findings to real-world context.

Weaknesses

- Minor: The societal and long-term impacts of risky sexual behavior among college students are not described.

2. Investigator(s):

Strengths

- PI Ray (Kentucky) is an early career investigator who has extensive experience coordinating brief alcohol intervention RCTs and working with community stakeholders to implement technology-based interventions.
- The expertise of the assembled research team is outstanding.
- A plan for coordination among organizations is outlined.

Weaknesses

- As noted in prior reviews, the overall team is rather large and multiple members of the team have overlapping expertise.

3. Innovation:

Strengths

- Hybrid effectiveness-implementation designs are underutilized in the brief alcohol intervention literature.
- Interventions to date have not included dynamic feedback linking personal trajectories of different behaviors.

Weaknesses

- None noted

4. Approach:

Strengths

- Investigators present preliminary data indicating that (a) the PFI is efficacious in this population in the short-term, (b) students are interested in feedback that updates based on their behavior, (c) students will complete the daily assessments, and (d) students will look at the feedback weekly, sometimes without being prompted. The proposed cross-tailored dynamic feedback modification represents a "modest" refinement to the existing intervention that is expected to improve its applicability in a previously-tested population; thus, this trial is appropriate for the hybrid effectiveness-implementation design.
- Use of retrospective and prospective assessment will allow investigators to examine effects across multiple modes of assessment.
- Investigators include a number of theoretically-based and high-impact secondary outcomes that will allow them to examine the fit of their theory and the societal impact of their intervention.

Weaknesses

- Investigators do not present the number of first-year students at each site or the expected rate of recruitment. It is unclear if recruitment of 300 participants in 12 months is feasible.
- Investigators will be underpowered to detect differences between the PFI+CDF vs PFI+GHI groups and, since no studies have examined the efficacy of the CDF, they have no evidence to support their claim that they will be sufficiently powered to detect this effect.
- Minor: Exclusion criteria are neglected in the proposal. It is unclear if investigators will include students with significant co-occurring mental health disorders or those who meet criteria for severe substance use disorders.
- Minor: It is not clear that the technology platform has been developed. This is considered a minor weakness, as the PI has a history of successful research collaboration with the tech team.

5. Environment:

Strengths

- The University of Kentucky and the University of North Texas have sufficient facilities and resources to undertake the proposed project.
- The Klein Buendel, Inc. provides unique resources in terms of intervention development.

Weaknesses

- It is unclear that institutions have sufficient participant flow to undertake the proposed RCT with a 13-month follow-up.

Study Timeline:

Strengths

- The timeline is generally organized and clear.

Weaknesses

- Participant flow is not described, so it is unclear if 12 months will be sufficient to recruit 300 heavy-drinking first-year students who are sexually active.
- There is a 6-month gap between the final Phase 2 follow-up assessment and the beginning of Phase 3 focus groups. The rationale for this is unclear.

Protections for Human Subjects:

Acceptable Risks and/or Adequate Protections

- Risks are considered and addressed.

Data and Safety Monitoring Plan (Applicable for Clinical Trials Only):

Acceptable

- An external Data Safety Monitoring Board will be appointed.

Inclusion Plans:

- Sex/Gender: Distribution justified scientifically
- Race/Ethnicity: Distribution justified scientifically
- For NIH-Defined Phase III trials, Plans for valid design and analysis: Scientifically acceptable
- Inclusion/Exclusion Based on Age: Distribution justified scientifically
- Inclusion plans are described and justified.

Vertebrate Animals:

Not Applicable (No Vertebrate Animals)

Biohazards:

Not Applicable (No Biohazards)

Resubmission:

- Investigators were responsive to critiques.

Resource Sharing Plans:

Acceptable

Budget and Period of Support:

Recommend as Requested

- No overlap identified.

THE FOLLOWING SECTIONS WERE PREPARED BY THE SCIENTIFIC REVIEW OFFICER TO SUMMARIZE THE OUTCOME OF DISCUSSIONS OF THE REVIEW COMMITTEE, OR REVIEWERS' WRITTEN CRITIQUES, ON THE FOLLOWING ISSUES:

PROTECTION OF HUMAN SUBJECTS: UNACCEPTABLE
No procedures are in place to address Title IX mandated reporting or follow-up contact with the participants regarding victimization experiences.

INCLUSION OF WOMEN PLAN: ACCEPTABLE

INCLUSION OF MINORITIES PLAN: ACCEPTABLE

INCLUSION ACROSS THE LIFESPAN: ACCEPTABLE

COMMITTEE BUDGET RECOMMENDATIONS: The budget was recommended as requested.

Footnotes for 1 R01 AA028246-01A1; PI Name: Ray, Anne Elizabeth

NIH has modified its policy regarding the receipt of resubmissions (amended applications).See Guide Notice NOT-OD-18-197 at https://grants.nih.gov/grants/guide/notice-files/NOT-OD-18-197.html. The impact/priority score is calculated after discussion of an application by averaging the overall scores (1-9) given by all voting reviewers on the committee and multiplying by 10. The criterion scores are submitted prior to the meeting by the individual reviewers assigned to an application, and are not discussed specifically at the review meeting or calculated into the overall impact score. Some applications also receive a percentile ranking. For details on the review process, see http://grants.nih.gov/grants/peer_review_process.htm#scoring.

MEETING ROSTER

Interventions to Prevent and Treat Addictions Study Section
Risk, Prevention and Health Behavior Integrated Review Group
CENTER FOR SCIENTIFIC REVIEW
IPTA
06/04/2020 - 06/05/2020

Notice of NIH Policy to All Applicants: Meeting rosters are provided for information purposes only. Applicant investigators and institutional officials must not communicate directly with study section members about an application before or after the review. Failure to observe this policy will create a serious breach of integrity in the peer review process, and may lead to actions outlined in NOT-OD-14-073 at https://grants.nih.gov/grants/guide/notice-files/NOT-OD-14-073.html and NOT-OD-15-106 at https://grants.nih.gov/grants/guide/notice-files/NOT-OD-15-106.html, including removal of the application from immediate review.

CHAIRPERSON(S)

WALTON, MAUREEN A, PHD, MPH
PROFESSOR
DEPARTMENT OF PSYCHIATRY
UNIVERSITY OF MICHIGAN
ANN ARBOR, MI 48109

MEMBERS

ALESSI, SHEILA MARIE, PHD *
ASSOCIATE PROFESSOR
DEPARTMENT OF PSYCHIATRY
CALHOUN CARDIOLOGY CENTER
UNIVERSITY OF CONNECTICUT HEALTH CENTER
FARMINGTON 06030

BERKEL, CADY, PHD *
ASSOCIATE RESEARCH PROFESSOR
REACH INSTITUTE
SOUTHWEST INTERDISCIPLINARY RESEARCH CENTER
ARIZONA STATE UNIVERSITY
TEMPE, AZ 85284

BERMAN, MITCHELL E, PHD *
PROFESSOR AND DEPARTMENT HEAD
DEPARTMENT OF PSYCHOLOGY
MISSISSIPPI STATE UNIVERSITY
MISSISSIPPI STATE, MS 39762

BERNSTEIN, STEVEN L, MD
PROFESSOR AND VICE CHAIR
DEPARTMENT OF EMERGENCY MEDICINE
YALE UNIVERSITY SCHOOL OF MEDICINE
NEW HAVEN, CT 06519

BRADIZZA, CLARA M, PHD
PROFESSOR
SCHOOL OF SOCIAL WORK
UNIVERSITY AT BUFFALO
STATE UNIVERSITY OF NEW YORK
BUFFALO, NY 14203

BUCKNER, JULIA D, PHD *
PROFESSOR AND DIRECTOR OF CLINICAL TRAINING
DEPARTMENT OF PSYCHOLOGY
LOUISIANA STATE UNIVERSITY
BATON ROUGE, LA 70803

COLEMAN-COWGER, VICTORIA HOPE, PHD
CLINICAL RESEARCH DIRECTOR
THE EMMES CORPORATION
ROCKVILLE, MD 20850

CROPSEY, KAREN L, PSYD
PROFESSOR
DEPARTMENT OF PSYCHIATRY AND BEHAVIORAL NEUROLOGY
UNIVERSITY OF ALABAMA AT BIRMINGHAM
BIRMINGHAM, AL 35294

DUNN, KELLY E., PHD *
ASSOCIATE PROFESSOR
DEPARTMENT OF PSYCHIATRY AND BEHAVIORAL SCIENCES
SCHOOL OF MEDICINE
JOHNS HOPKINS UNIVERSITY
BALTIMORE, MD 21224-6823

FRIEDMANN, PETER D, MD, MPH
PROFESSOR
DEPARTMENT OF MEDICINE
UNIVERSITY OF MASSACHUSETTS
MEDICAL SCHOOL - BAYSTATE
SPRINGFIELD, MA 01107

GRANT, SEAN, PHD *
ASSISTANT PROFESSOR
RICHARD M. FAIRBANKS SCHOOL OF PUBLIC HEALTH
INDIANA UNIV-PURDUE UNIV AT INDIANAPOLIS
INDIANAPOLIS, IN 46202

GRYCZYNSKI, JAN, PHD
SENIOR RESEARCH SCIENTIST
FRIENDS RESEARCH INSTITUTE
BALTIMORE, MD 21201

HETTEMA, JENNIFER ELIN, PHD *
ASSOCIATE PROFESSOR
DEPARTMENT OF FAMILY
AND COMMUNITY MEDICINE
UNIVERSITY OF NEW MEXICO SCHOOL OF MEDICINE
ALBUQUERQUE 87131

HILL, KEVIN P., MD *
ASSOCIATE PROFESSOR OF PSYCHIATRY
HARVARD MEDICAL SCHOOL
BOSTON, MA 02215

HITSMAN, BRIAN L, PHD
ASSOCIATE PROFESSOR
DEPARTMENT OF PREVENTIVE MEDICINE
FEINBERG SCHOOL OF MEDICINE
NORTHWESTERN UNIVERSITY
CHICAGO, IL 60611

INGERSOLL, KAREN S, PHD *
PROFESSOR
DEPARTMENT OF PSYCHIATRY
AND NEUROBEHAVIORAL SCIENCES
UNIVERSITY OF VIRGINIA
CHARLOTTESVILLE, VA 22911

KAHLER, CHRISTOPHER W., PHD *
PROFESSOR AND CHAIR
DEPARTMENT OF BEHAVIORAL
AND SOCIAL SCIENCES
CENTER FOR ALCOHOL AND ADDICTION STUDIES
BROWN UNIVERSITY SCHOOL OF PUBLIC HEALTH
PROVIDENCE, RI 02912

KIM-MOZELESKI, JIN E., PHD *
ASSISTANT PROFESSOR
DEPARTMENT OF POPULATION AND
QUANTITATIVE HEALTH SCIENCES
CASE WESTERN RESERVE UNIVERSITY
CLEVELAND, OH 44106

LARSON, MARY JO, PHD
SENIOR SCIENTIST
INSTITUTE FOR BEHAVIORAL HEALTH
HELLER SCHOOL FOR SOCIAL POLICY AND MANAGEMENT
BRANDEIS UNIVERSITY
WALTHAM, MA 02454

LITT, DANA M, PHD *
ASSOCIATE PROFESSOR
DEPARTMENT OF HEALTH BEHAVIOR AND HEALTH
SYSTEMS
SCHOOL OF PUBLIC HEALTH SYSTEMS
UNIVERSITY OF NORTH TEXAS
FORT WORTH 76107

LOVEJOY, TRAVIS IAN, MPH, PHD *
ASSOCIATE PROFESSOR
DEPARTMENT OF PSYCHIATRY
SCHOOL OF MEDICINE
OREGON HEALTH AND SCIENCE UNIVERSITY
PORTLAND, OR 97239

MAHABEE-GITTENS, E. MELINDA, MD
PROFESSOR
DIVISION OF EMERGENCY MEDICINE
CINCINNATI CHILDREN'S HOSPITAL MEDICAL CENTER
CINCINNATI, OH 45229

MAISTO, STEPHEN A, PHD *
PROFESSOR
DEPARTMENT OF PSYCHOLOGY
SYRACUSE UNIVERSITY
SYRACUSE, NY 13244

MCGOVERN, MARK P, PHD
PROFESSOR
DEPARTMENT OF PSYCHIATRY
AND BEHAVIORAL SCIENCES
STANFORD UNIVERSITY
PALO ALTO, CA 93404

MENDELSON, TAMAR, PHD
PROFESSOR
DEPARTMENT OF MENTAL HEALTH
JOHNS HOPKINS BLOOMBERG SCHOOL OF PUBLIC HEALTH
BALTIMORE, MD 21205

MILLER, MARY ELIZABETH, PHD *
ASSISTANT PROFESSOR
DEPARTMENT OF PSYCHIATRY
UNIVERSITY OF MISSOURI
COLUMBIA, MO 65211

MONTGOMERY, LATRICE, PHD *
ASSISTANT PROFESSOR
ADDICTION SCIENCES DIVISION
DEPARTMENT OF PSYCHIATRY
AND BEHAVIORAL NEUROSCIENCE
UNIVERSITY OF CINCINNATI COLLEGE OF MEDICINE
CINCINNATI, OH 45229

NOONAN, DEVON, PHD *
ASSOCIATE PROFESSOR
SCHOOL OF NURSING
DUKE UNIVERSITY
DURHAM, NC 27710

OLIVETO, ALISON, PHD
PROFESSOR AND VICE CHAIR FOR RESEARCH
DEPARTMENT OF PSYCHIATRY
UNIVERSITY OF ARKANSAS FOR MEDICAL SCIENCES
LITTLE ROCK, AR 72205

OSILLA, KAREN C, PHD
SENIOR BEHAVIORAL SCIENTIST
RAND CORPORATION
SANTA MONICA, CA 90404

PARROTT, DOMINIC, PHD *
PROFESSOR
DEPARTMENT OF PSYCHOLOGY
GEORGIA STATE UNIVERSITY
ATLANTA, GA 30302

PIPER, MEGAN E, PHD
ASSOCIATE PROFESSOR
DEPARTMENT OF MEDICINE
UNIVERSITY OF WISCONSIN-MADISON
MADISON, WI 53711

SCHEUERMANN, TANEISHA SHANI, PHD *
ASSISTANT PROFESSOR
DEPARTMENT OF PREVENTIVE MEDICINE AND
PUBLIC HEALTH
UNIVERSITY OF KANSAS MEDICAL CENTER
KANSAS CITY, KS 66160

STEIN, MICHAEL D, MD
PROFESSOR AND CHAIR
DEPARTMENT OF HEALTH LAW, POLICY AND MANAGEMENT
BOSTON UNIVERSITY SCHOOL OF PUBLIC HEALTH
BOSTON, MA 02118

STEINBERG, MARC L, PHD *
ASSOCIATE PROFESSOR
DIVISION OF ADDICTION PSYCHIATRY
ROBERT WOOD JOHNSON MEDICAL SCHOOL
RUTGERS, THE STATE UNIVERSITY OF NEW JERSEY
NEW BRUNSWICK, NJ 08901

TINDLE, HILARY A, MD, MPH
ASSOCIATE PROFESSOR
DIVISION OF INTERNAL MEDICINE AND PUBLIC HEALTH
VANDERBILT UNIVERSITY MEDICAL CENTER
NASHVILLE, TN 37203

TSOH, JANICE Y, PHD
PROFESSOR
DEPARTMENT OF PSYCHIATRY
LANGLEY PORTER PSYCHIATRIC INSTITUTE
UNIVERSITY OF CALIFORNIA SAN FRANCISCO
SAN FRANCISCO, CA 94143

TSUI, JUDITH, MD, MPH *
ASSOCIATE PROFESSOR OF MEDICINE
DIVISION OF GENERAL INTERNAL MEDICINE
UNIVERSITY OF WASHINGTON SCHOOL OF MEDICINE
HARBORVIEW MEDICAL CENTER
SEATTLE, WA 98122

VELASQUEZ, MARY M, PHD
CENTENNIAL PROFESSOR AND DIRECTOR
HEALTH BEHAVIOR RESEARCH
AND TRAINING INSTITUTE
STEVE HICKS SCHOOL OF SOCIAL WORK
UNIVERSITY OF TEXAS AT AUSTIN
AUSTIN, TX 78712

VINCI, CHRISTINE, PHD *
ASSISTANT MEMBER
DEPARTMENT OF HEALTH OUTCOMES AND BEHAVIOR
MOFFITT CANCER CENTER
TAMPA, FL 33612

WINSTANLEY, ERIN L, PHD *
ASSOCIATE PROFESSOR
DEPARTMENT OF BEHAVIORAL MEDICINE AND PSYCHIATRY
WEST VIRGINIA UNIVERSITY, SCHOOL OF MEDICINE
MORGANTOWN, WV 26506

YI, RICHARD, PHD *
PROFESSOR
DEPARTMENT OF PSYCHOLOGY
COFRIN LOGAN CENTER FOR ADDICTION RESEARCH AND
TREATMENT
UNIVERSITY OF KANSAS
LAWRENCE, KS 66045

SCIENTIFIC REVIEW OFFICER

MINTZER, MIRIAM, PHD
SCIENTIFIC REVIEW OFFICER
CENTER FOR SCIENTIFIC REVIEW
NATIONAL INSTITUTES OF HEALTH
BETHESDA, MD 20892

EXTRAMURAL SUPPORT ASSISTANT

FAYEMIWO, TOLU, MS
EXTRAMURAL SUPPORT ASSISTANT
CENTER FOR SCIENTIFIC REVIIEW
NATIONAL INSTITUTES OF HEALTH
BETHESDA, MD 20892

* Temporary Member. For grant applications, temporary members may participate in the entire meeting or may review only selected applications as needed.

Consultants are required to absent themselves from the room during the review of any application if their presence would constitute or appear to constitute a conflict of interest.
